# Optimizing thermoelectric performance of carbon-doped h-BN monolayers through tuning carrier concentrations and magnetic field

**DOI:** 10.1038/s41598-023-46116-w

**Published:** 2023-11-10

**Authors:** Somayeh Behzad, Raad Chegel

**Affiliations:** 1 Department of Engineering Physics, Kermanshah University of Technology, Kermanshah, Iran; 2 Department of Physics, Faculty of Science, Malayer University, Malayer, Iran

**Keywords:** Electronic properties and materials, Semiconductors, Electronic properties and materials, Two-dimensional materials, Two-dimensional materials

## Abstract

The thermoelectric properties of carbon-doped monolayer hexagonal boron nitride (h-BN) are studied using a tight-binding model employing Green function approach and the Kubo formalism. Accurate tight-binding parameters are obtained to achieve excellent fitting with Density Functional Theory results for doped h-BN structures with impurity type and concentration. The influence of carbon doping on the electronic properties, electrical conductivity, and heat capacity of h-BN is studied, especially under an applied magnetic field. Electronic properties are significantly altered by doping type, concentration, and magnetic field due to subband splitting, merging of adjacent subbands, and band gap reduction. These modifications influence the number, location, and magnitude of DOS peaks, generating extra peaks inside the band gap region. Heat capacity displays pronounced dependence on both magnetic field and impurity concentration, exhibiting higher intensity at lower dopant levels. Electrical conductivity is increased by double carbon doping compared to single doping, but is reduced at high magnetic fields because of high carrier scattering. The electronic figure of merit ZT increases with lower impurity concentration and is higher for CB versus CN doping at a given field strength. The power factor can be improved by increasing magnetic field and decreasing doping concentration. In summary, controlling doping and magnetic field demonstrates the ability to effectively engineer the thermoelectric properties of monolayer h-BN.

## Introduction

Two-dimensional (2D) materials are a class of emerging materials with unique electronic, optical, mechanical, and thermal properties that have the potential to revolutionize a wide range of technologies, including electronics, energy storage, and sensors^1^. 2D materials are being used to create a wide range of new devices, including transistors, sensors, batteries, and supercapacitors^2,3^. They are also being used to develop new methods for catalysis and energy production. The potential applications of 2D materials are vast, and they are sure to play a major role in the future of science and technology^4–7^.

The synthesis of doped 2D materials is more difficult compared to pure 2D materials. Doping can be achieved through several methods like mechanical exfoliation, surface functionalization, vapor deposition, plasma processing, absorption, electrochemical techniques, and thermal evaporation. Each of these methods has its own strengths and limitations^8^. Mechanical exfoliation is suitable for obtaining ultrathin, high-quality 2D materials due to weak van der Waals forces^9,10^, but it lacks control over layer thickness^8^. Surface functionalization allows various compositions of 2D functional units on few-layered 2D materials but needs improved stability^8^. Vapor deposition techniques like PVD and CVD are widely used to synthesize large-area, high-quality doped 2D materials^11^. The thickness and dopant concentration can be precisely controlled during growth^12^. The process of electrochemical intercalation has been utilized to dope 2DMs and generate a wide variety of superlattices with customized molecular structures, interlayer spacings, and electronic and optical characteristics^13^.

Graphene, a flat single layer of carbon atoms structured in a honeycomb lattice, has emerged as the most widely studied two-dimensional (2D) material^14^. In graphene, the valence and conduction bands meet at the Dirac point, resulting in a zero band gap and linear dispersion of the energy bands near this point^15^. At this point, known as the Dirac point, graphene hosts massless fermion particles and exhibits strange electronic properties^16^. In the vicinity of the Dirac point, the graphene band structure exhibits linear behavior with respect to the momentum, which gives graphene its unique electrical and optical properties^17^. Many studies have shown that accurate control of the Dirac point voltage is crucial for developing new graphene-based nanoelectronic and optoelectronic devices with improved performance and functionality^18,19^. The Dirac point voltage can be controlled by doping graphene with impurities, but this can also change the other properties of graphene^20^.

Unlike single-element graphene-like materials like Silicene and Germanene which have buckled honeycomb lattices and zero band gaps^21^, dual-element graphene analogs such as BN, SiC, BeO and BP have planar honeycomb structures and nonzero band gaps^22–24^. When an electric field is applied perpendicular to the non-planar materials such as Silicene and Germanene, their band gaps can be tuned from zero to non-zero values, which allows their electronic behavior to be switched from semi-metallic to semiconducting^25–27^. This behavior is unlike planar materials which are insensitive to vertical bias voltage and their electronic properties can be optimized for different applications through techniques like doping and strain engineering^22,28,29^.

Two-dimensional Hexagonal boron nitride (h-BN) is a two-dimensional material that made up of boron (B) and nitrogen (N) atoms arranged in a hexagonal pattern, similar to graphene. Same to the Graphene, in recent years, there has been a lot of research on the different properties of h-BN^28,30^. This interest stems from h-BN’s highly stable structure, mechanical properties and good thermal conductivity^30–33^. However, unlike graphene, h-BN is an insulator with a wide band gap of around 4 eV. This makes h-BN transparent to visible light and makes it a promising material for optoelectronic applications, such as solar cells and lasers. In addition to graphene like h-BN, there are also a number of h-BN nanostructures, such as nanotubes and nanoribbons which are being investigated for a variety of applications such as nanoscale transistors, sensors, solar cells and lasers. The unique properties of h-BN, such as its insulating properties, similar lattice constant and structure to graphene, make it a good choice for use as a charge leakage barrier layer, to improve the stability and quality of graphene’s electrical properties for future electronics^34^.

The unique optical properties of h-BN, such as its lack of visible light absorption^35,36^ and its ability to emit and detect deep ultraviolet (UV) light^37^, make it suitable for useful applications in optical storage. h-BN has been shown to have promising gas sensing properties. It has been investigated for different gas molecules such as NO_2_, NO, NH_3_, and CO^38,39^.

The large band gap of h-BN can be modulated and controlled through external parameters including strain^40–43^, an applied external electric field^44,45^, introducing impurities^46–49^, and formation of multilayers in the presence of a bias voltage. The purpose of this study is to investigate the effects of carbon impurities on the thermoelectric properties of h-BN structures. Carbon was chosen as the dopant atom because it has a similar atomic radius to boron and nitrogen, making it a good candidate for substitutional impurities in the BN lattice. Note that carbon is not the only dopant atom that can be doped into h-BN. Other atoms such as silicon^48^ and oxygen^50^ can also be doped into h-BN and decrease its band gap. For example, silicon doping can decrease the wide band gap of h-BN to 1.24 eV and 0.84 eV when it is substituted for boron and nitrogen atoms, respectively^48^. In addition to band gap tuning in BN with C doping, its absorption characteristics^51,52^ and the poor performance of h-BN-based photodetectors^53^ can also be influenced by C doping. In addition to the above mentioned structures, 2D carbon nitride nanostructures such as C6N7 have also been extensively studied. These materials have been shown to be mechanically stable with semiconducting properties^54,55^.

The thermal properties of the h-BN are of interest and have been investigated in several studies. At room temperature, the thermal conductivity of monolayer h-BN is about 600 W/mK which is lower than graphene’s thermal conductivity between 3500 and 5300 W/mK strength^56,57^. The number of layers in h-BN structures also affects their thermal conductivity, with thicker layers having lower thermal conductivity^58^. For instance, five-layer BN possesses a thermal conductivity around 250 W/mK, , which is nearly comparable to the thermal conductivity of bulk BN^59^.

Multilayer structures composed of graphene and BN can exhibit enhanced thermal conductivity, as demonstrated in bilayer graphene/BN^23^. Besides graphene and BN, BCN structures have also been studied theoretically^60^ and found to exhibit high thermal conductivity. The thermal conductivity of BCN structures is less temperature-dependent than graphene or BN, and it decreases with compressive strain and increases with tensile strain^61^. BC_2_N is a more stable form of BCN than BCN, and it has higher thermal conductivity than BCN, especially at high temperatures and under strain^62^. This makes BC_2_N a particularly attractive material for high-performance thermal management applications.

While BN nanostructures have been extensively studied for their electronic properties, investigations into their thermal characteristics remain lacking. In particular, the thermal properties of carbon-doped h-BN under magnetic fields represent an open area of research. This work examines the impact of impurity concentration on the thermoelectric properties of carbon-doped boron nitride. The theoretical framework employs the Kubo-Greenwood formula based on Green’s function formalism to provide fundamental insights into how doping and magnetic fields influence heat transport in these materials. This work aims to advance the limited understanding of the thermal properties of doped BN nanostructures. These findings can stimulate future research on engineering h-BN with optimal thermoelectric performance through impurity doping for thermoelectric generators, and thermal management.

## Theoretical tight binding formalism

**Figure 1:**
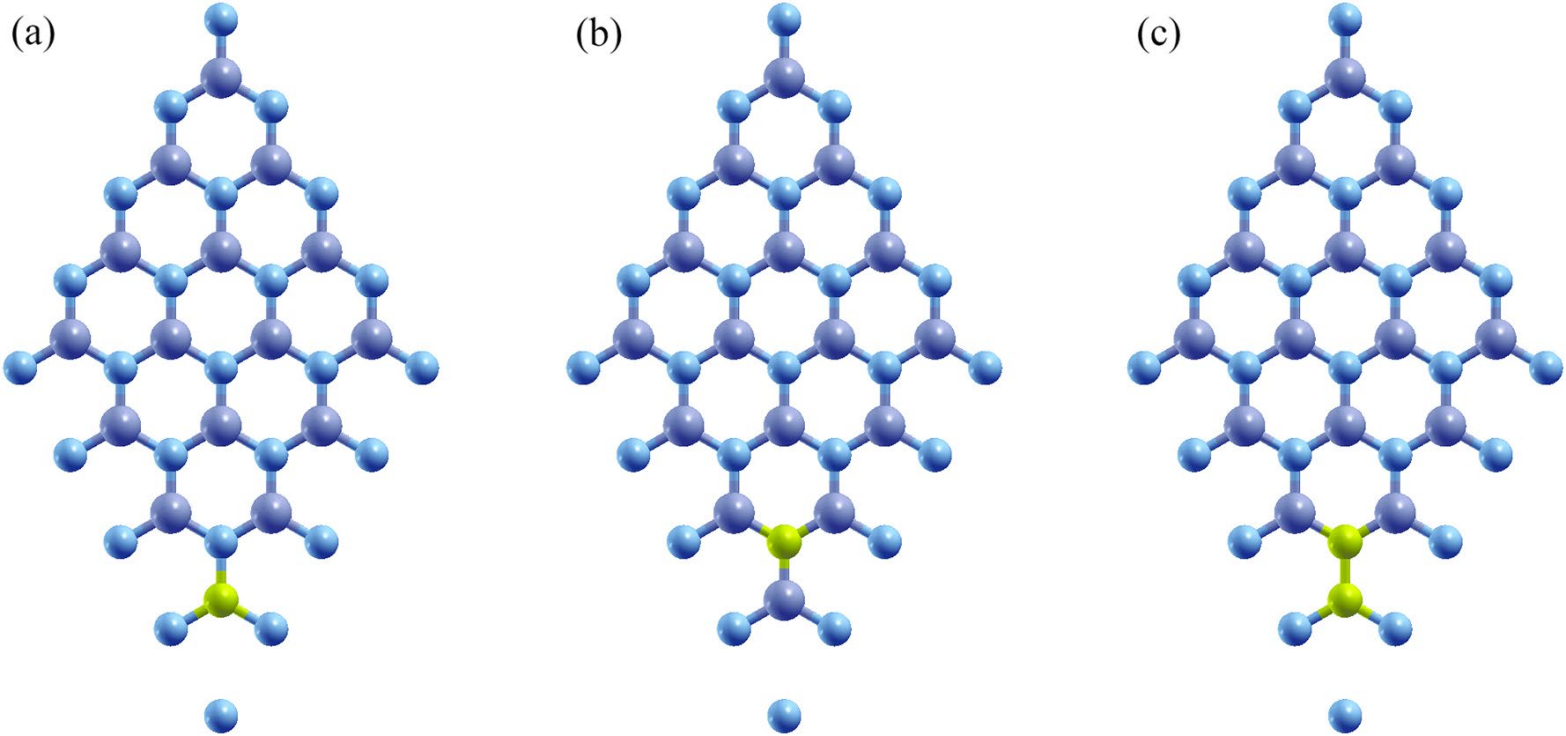
Schematic picture of the atomic structure for the N_4_-doped h-BN with (**a**) C_B_, (**b**) C_N_ and (**c**) C_BN_.

The Hamiltonian matrix should be calculated to obtain the band structure of the h-BN structure. For the pure h-BN case, the unit cell contains two atoms and the Hamiltonian matrix is described with the 2 × 2 matrix^63^. When impurities are added to the structure, controlling the impurity concentrations is essential and can be defined based on the ratio of impurity atoms to the all atoms. So, by increasing the number of unit cells in the structure, different concentrations of impurities can be modeled. In order to model different impurity concentrations, the pristine h-BN unit cell can be extended along the monolayer’s primitive lattice vectors a1 and a_2_ to create a supercell of dimensions (Na_1_,Na_2_), where N is the number of unit cells in each direction. The supercell of pure h-BN structure with N unit cells has $N^{2}$ boron type and $N^{2}$ nitrogen type atoms. For a supercell containing m impurity atoms, the impurity concentration is given by $\frac{m}{{2N^{2} }}$. By increasing the supercell size N, different impurity concentrations can be achieved while keeping the number of impurities m fixed. In this study, the structure with one and two impurity atoms and three (N_3_), four (N_4_) and five (N_5_) unit cells have been selected and these supercells correspond to impurity concentration lies in between 2 and 5% for single impurity and 4–10% for double impurity, respectively. The schematic views of supercells with N = 4 are shown in Fig. 1 and the required tight binding parameters for these structures are obtained from DFT calculations [Supporting file]. For the selected structures, the total Hamiltonian in second quantization is given by:

1 $${\text{H}}_{0} = \mathop \sum \limits_{i,\alpha ,\sigma } \left[ {\upvarepsilon _{i}^{\sigma } \left( \alpha \right)} \right]c_{i,\alpha }^{\dag \sigma } c_{i,\alpha }^{\sigma } + \mathop \sum \limits_{i,j,\sigma ,\alpha ,\beta } {\text{t}}_{ij}^{{\alpha ,\upbeta }} (c_{i,\alpha }^{\dag \sigma } c_{j,\beta }^{\sigma } + h.c.) + \mathop \sum \limits_{i,\sigma ,\alpha } \Pi \left( {B_{0} } \right)\left( {c_{i,\alpha }^{\dag \uparrow } c_{i,\alpha }^{ \downarrow } + c_{i,\alpha }^{\dag \downarrow } c_{i,\alpha }^{ \uparrow } } \right)$$

where the first and second parts are correspond to the local onsite energies and interaction between neighbor atoms, respectively. The $C_{i,\alpha }^{\dag \sigma }$ and $C_{i,\alpha }^{\sigma }$ represent the creation and annihilation operators with spin σ for $\alpha$-th atom type in i-th unit cell. The onsite energy for α-th type with spin σ in the primitive unit cell $i$, is shown with the $\upvarepsilon _{i}^{\sigma } \left( \alpha \right)$.

Calculating the Hamiltonian matrix required specifying tight binding parameters such as the hopping integrals between neighboring atoms and the on-site energies for each atom type. The relevant atom types are boron and nitrogen, which form the pristine h-BN structure, and carbon atoms as impurities.

DFT calculations were performed to obtain the band structure of carbon-doped h-BN structures with different impurity concentrations (Fig. S1). By fitting the tight binding band structure to the corresponding DFT results, the required tight binding hopping integrals and on-site energies were extracted for each atom type. This obtained tight binding parameters that can reproduce the DFT band structure of carbon-doped h-BN using the values given in Table 1.

**Table 1:** Tight binding parameters for carbon-doped h-BN.

Hopping (eV)	$t_{B - N} = 2.58$	$t_{B - C} = 2.41$	$t_{N - C} = 2.25$	$t_{C - C} = 1.95$
On-site energy (eV)	$\varepsilon_{C} = 0$	$\varepsilon_{B} = 1.87$	$\varepsilon_{N} = - 2.13$	-

The table provides the optimized tight binding parameters (in eV), including on-site energies and hopping integrals between different atom pairs. These parameters were obtained by fitting the tight binding model to DFT band structures.

By applying the magnetic field $B_{T} = \sigma g\mu_{B} B_{0}$, the Hamiltonian matrix becomes spin dependent and it labeled by $H^{\sigma } \left( {\varvec{k}} \right)$ for the spin up and down. The calculations in this work utilize a rescaled magnetic field value of $B_{T} = \sigma g\mu_{B} B_{0}$. The matrix form for Hamiltonian $H\left( {\varvec{k}} \right)$ in the ${\varvec{k}}$ space can be obtained with the Fourier transformation for the creation and annihilation fermion operators as:

2 $$C_{i,\alpha }^{\dag \sigma } = \frac{1}{\sqrt N }\mathop \sum \limits_{k} C_{k,\alpha }^{\dag \sigma } e^{{ - i{\varvec{k}}.{\varvec{R}}_{i}^{\alpha } }} \quad C_{i,\alpha }^{\sigma } = \frac{1}{\sqrt N }\mathop \sum \limits_{k} C_{k,\alpha }^{\sigma } e^{{i{\varvec{k}}.{\varvec{R}}_{i}^{\alpha } }}$$

The BN doped structure with $N$ unit cell, has $4N^{2} \times 4N^{2}$ Hamiltonian matrix and its band structure $E^{\sigma } \left( {\varvec{k}} \right)$ can be obtained by solving the Schrodinger equation $H^{\sigma } \left( {\varvec{k}} \right)C\left( {\varvec{k}} \right) = E^{\sigma } \left( {\varvec{k}} \right)C\left( {\varvec{k}} \right)$ where the wave vector ${\varvec{k}} \equiv \left( {k_{x} ,k_{y} } \right)$ is surrounded in the first Brillouin zone.

The density of states (DOS) is obtained from imaginary part of Green’s function with $\frac{ - 1}{\pi }Im[{\varvec{G}}_{lj} \left( E \right)]$ and the Green function is defined by $G_{ij}^{\alpha \beta } \left( \tau \right) = - \hat{T}\hat{C}_{{i{\upalpha }}}^{\sigma } \left( \tau \right)\hat{C}_{{j{\upbeta }}}^{\dag \sigma } \left( \tau \right)\left( 0 \right)$ where τ is the imaginary time and $\hat{T}$ is the time ordering operator.

By applying the Heisenberg equation for the $\hat{C}_{{i{\upalpha }}}^{\sigma }$ and $\hat{C}_{{j{\upbeta }}}^{\dag \sigma }$ operators and by using the imaginary time Fourier transformation ${\varvec{G}}_{lj} \left( \tau \right) = \frac{1}{{k_{B} T}}\mathop \sum \limits_{l} e^{{i\omega_{n} \tau }} {\varvec{G}}_{lj} \left( {i\omega_{n} } \right)$ with $i\omega_{n} \to E \equiv E + i0^{ + }$, the equation of motion for the electron is obtained as:

3 $$\mathop \sum \limits_{s} \left[ {\left( {E{\varvec{I}} - {{\varvec{\Omega}}}\left( {B_{T} } \right)} \right)\delta_{is} + {\varvec{t}}_{is} } \right]{\varvec{G}}_{sj} \left( E \right) = {\varvec{I}}\delta_{ij}$$

where $\omega_{n} = \frac{{\left( {n + 1} \right)\pi }}{{k_{B} T}}$ is the fermionic Matsubara frequencies, ${\varvec{t}}_{il}$ is the hopping integral matrix and ${{\varvec{\Omega}}}\left( {B_{T} } \right)$ is the on-site energy matrixe in the presence of magnetic field, respectively. The DOS is obtained from imaginary part of Green’s function with $\frac{ - 1}{\pi }Im[{\varvec{G}}_{lj} \left( E \right)]$.

The thermal properties of doped graphene are calculated using the Kubo-Greenwood formula, and this formalism requires calculating the spectral function ${\varvec{A}}\left( {{\varvec{k}},\varepsilon } \right) = - 2Im\hat{G}\left( {k,\varepsilon } \right)$ which is related to the DOS. In the presence of the temperature gradient $\nabla {\text{T}}$, the electrical charge current $J^{e}$ and thermal heat current $J^{Q}$ are defined in terms of the transport coefficients^64^

4 $$\left( {\begin{array}{*{20}c} {J_{1} \equiv J^{e} } \\ {J_{2} \equiv J^{Q} } \\ \end{array} } \right) = \left( {\begin{array}{*{20}c} {{\Upsilon }_{11} } & {{\Upsilon }_{12} } \\ {{\Upsilon }_{21} } & {{\Upsilon }_{22} } \\ \end{array} } \right)\left( {\begin{array}{*{20}c} E \\ {\nabla {\text{T}}} \\ \end{array} } \right)$$

and the ${\Upsilon }_{mn}$ coefficients are obtained in terms of the correlation function between $J^{e}$ and $J^{Q}$ current operators as^65^:

5 $$\left( {\begin{array}{*{20}c} {{\Upsilon }_{11} } \\ {{\Upsilon }_{12} } \\ {{\Upsilon }_{22} } \\ \end{array} } \right) = \frac{i}{\beta \omega } \mathop {\lim }\limits_{{i\omega_{n} \to \omega + i0^{ + } }} \mathop \smallint \limits_{0}^{\beta } d\tau e^{{i\omega_{n} \tau }} T_{\tau } \left( {\begin{array}{*{20}c} {\hat{J}^{e} \left( \tau \right)\hat{J}^{e} \left( 0 \right)} \\ {\hat{J}^{e} \left( \tau \right)\hat{J}^{Q} \left( 0 \right)} \\ {\hat{J}^{Q} \left( \tau \right)\hat{J}^{Q} \left( 0 \right)} \\ \end{array} } \right)$$

The charge current operator function $\hat{J}^{e}$ and the heat current operator $\hat{J}^{Q}$ can be defined as^64^

6a $$\hat{J}^{e} = \mathop \sum \limits_{k} \mathop \sum \limits_{p} \nu_{k}^{\left( p \right)} c_{k}^{\dag \left( p \right)} c_{k}^{\left( p \right)}$$

7b $$\hat{J}^{Q} = \mathop \sum \limits_{k} \mathop \sum \limits_{p} E_{p} \left( {{\varvec{k}},B_{T} } \right) \nu_{k}^{\left( p \right)} c_{k}^{\dag \left( p \right)} c_{k}^{\left( p \right)}$$

where $\nu_{k}^{\left( p \right)} = \partial_{k} E_{p} \left( {{\varvec{k}},B_{T} } \right)$ is the velocity operator for the p-th Hamiltonian eigenvalues in presence of the magnetic field. By using the Wick’s theorem based on the Green function $G_{p} \left( {k,\tau } \right) = - T_{\tau } { }c_{k}^{\dag \left( p \right)} \left( \tau \right){ }c_{k}^{\left( p \right)} \left( 0 \right)$, the correlation function parts can be written as:

8a $$T_{\tau } { }\hat{J}^{e} \left( \tau \right)\hat{J}^{e} \left( 0 \right) = \mathop \sum \limits_{k,p} \left( {{ }\nu_{k}^{\left( p \right)} } \right)^{2} G_{p} \left( {k,\tau } \right)G_{p} \left( {k, - \tau } \right)$$

8b $$T_{\tau } \hat{J}^{e} \left( \tau \right)\hat{J}^{Q} \left( 0 \right) = \mathop \sum \limits_{k,p} E_{p} \left( {k,B_{T} } \right)\left( { \nu_{k}^{\left( p \right)} } \right)^{2} G_{p} \left( {k,\tau } \right)G_{p} \left( {k, - \tau } \right)$$

8c $$T_{\tau } \hat{J}^{Q} \left( \tau \right)\hat{J}^{Q} \left( 0 \right) = \mathop \sum \limits_{k,p} \left( {E_{p} \left( {k,B_{T} } \right)} \right)^{2} \left( { \nu_{k}^{\left( p \right)} } \right)^{2} G_{p} \left( {k,\tau } \right)G_{p} \left( {k, - \tau } \right)$$

By using the Fourier transform of Green function $\left[ {G_{p} \left( {k,\tau } \right) = \mathop \sum \limits_{m} e^{{ - i\omega_{m} \tau }} G_{p} \left( {k,i\omega_{m} } \right)} \right]$, the transport coefficients Eq. (4) can be expressed as:

9 $${\Upsilon }_{{{\text{ij}}}} \left( {{\text{T}},{\Pi }} \right) = \frac{i}{{\beta^{2} \omega }}{ }\mathop {{\text{lim}}}\limits_{{i\omega_{n} \to \omega + i0^{ + } }} { }\mathop \sum \limits_{k,p} \mathop \sum \limits_{m} \left( {E_{p} \left( {{\varvec{k}},{\Pi }} \right)} \right)^{{{\text{i}} + {\text{j}} - 2}} { }\left( {{ }\nu_{k}^{\left( p \right)} } \right)^{2} G_{p} \left( {k,i\omega_{m} } \right)G_{p} \left( {k,i\omega_{n} + i\omega_{m} } \right)$$

which the $G_{p} \left( {k,i\omega_{m} } \right) = \mathop \smallint \limits_{ - \infty }^{ + \infty } \frac{d\varepsilon }{{2\pi }}{ }\frac{{A_{p} \left( {k,\varepsilon } \right)}}{{i\omega_{m} - \varepsilon }}$ is related to the spectral function $A_{p} \left( {k,\varepsilon } \right)$. Finally, using the Matsubara frequency summation and Eq. (9), the transport coefficient ${\Upsilon }_{mn}$ obtained from the following equation:

10 $${\Upsilon }_{{{\text{ij}}}} \left( {{\text{T}},{\Pi }} \right) = \frac{1}{\beta }{ }\mathop \smallint \limits_{ - \infty }^{ + \infty } \left[ {\frac{\partial f\left( \varepsilon \right)}{{\partial \varepsilon }}} \right]\frac{d\varepsilon }{{2\pi }}{ }\mathop \sum \limits_{k,p} \left( {E_{p} \left( {{\varvec{k}},B_{T} } \right)} \right)^{{{\text{i}} + {\text{j}} - 2}} \left( {{ }\nu_{k}^{\left( p \right)} A_{p} \left( {k,\varepsilon } \right)} \right)^{2}$$

where the Fermi–Dirac distribution function is shown by the $f\left( {\varepsilon ,T} \right) = \left[ {1 + exp\left( {\varepsilon /k_{B} T} \right)} \right]^{ - 1}$.

The temperature dependence of the electrical conductivity $\sigma \left( {\text{T}} \right)$ is proportional to ${\Pi }_{11}$ and is obtained by^66^:

11 $$\sigma \left( {{\text{T}},B_{T} } \right) = \frac{1}{\beta }{ }\mathop \smallint \limits_{ - \infty }^{ + \infty } \left[ {\frac{\partial f\left( \varepsilon \right)}{{\partial \varepsilon }}} \right]\frac{d\varepsilon }{{2\pi }}{ }\mathop \sum \limits_{k,p} \left( {{ }\nu_{k}^{\left( p \right)} A_{p} \left( {k,\varepsilon } \right)} \right)^{2}$$

By using the obtained equations for the electrical and thermal conductivity, the temperature dependence of the Lorenz number and thermoelectric figure-of-merit can be defined in terms of the transport coefficients ${\Upsilon }_{{{\text{ij}}}}$, as^66^:

12a $$ZT\left( {\text{T}} \right) = \frac{{[{\Upsilon }_{12} ]^{2} }}{{{\Upsilon }_{11} {\Upsilon }_{22} - [{\Upsilon }_{12} ]^{2} }}$$

12b $${\text{PF}}\left( T \right) \equiv \frac{{[{\Upsilon }_{12} ]^{2} }}{{{\text{T}}^{2} {\Upsilon }_{11} }}$$

The heat capacity ${\text{C}}\left( {\text{T}} \right)$ is the response of the total internal energy $U_{0} = \mathop \smallint \limits_{ - \infty }^{\infty } \varepsilon { }f\left( {\varepsilon ,T} \right){ }D\left( \varepsilon \right){ }d\varepsilon$ of N electrons to a temperature changes [${\text{C}}\left( {\text{T}} \right) = \frac{{\partial U_{0} }}{\partial T}$] and using the DOS spectrum, the electronic heat capacity ${\text{C}}\left( {\text{T}} \right)$ in terms of the temperature, it has been defined as^67^:

13 $${\text{C}}\left( {\text{T}} \right) = \mathop \smallint \limits_{ - \infty }^{\infty } \varepsilon \left[ {\frac{{\partial f\left( {\varepsilon ,T} \right)}}{\partial T}} \right]D\left( \varepsilon \right){ }d\varepsilon$$

## Results and discussions

### Electronic properties

**Figure 2:**
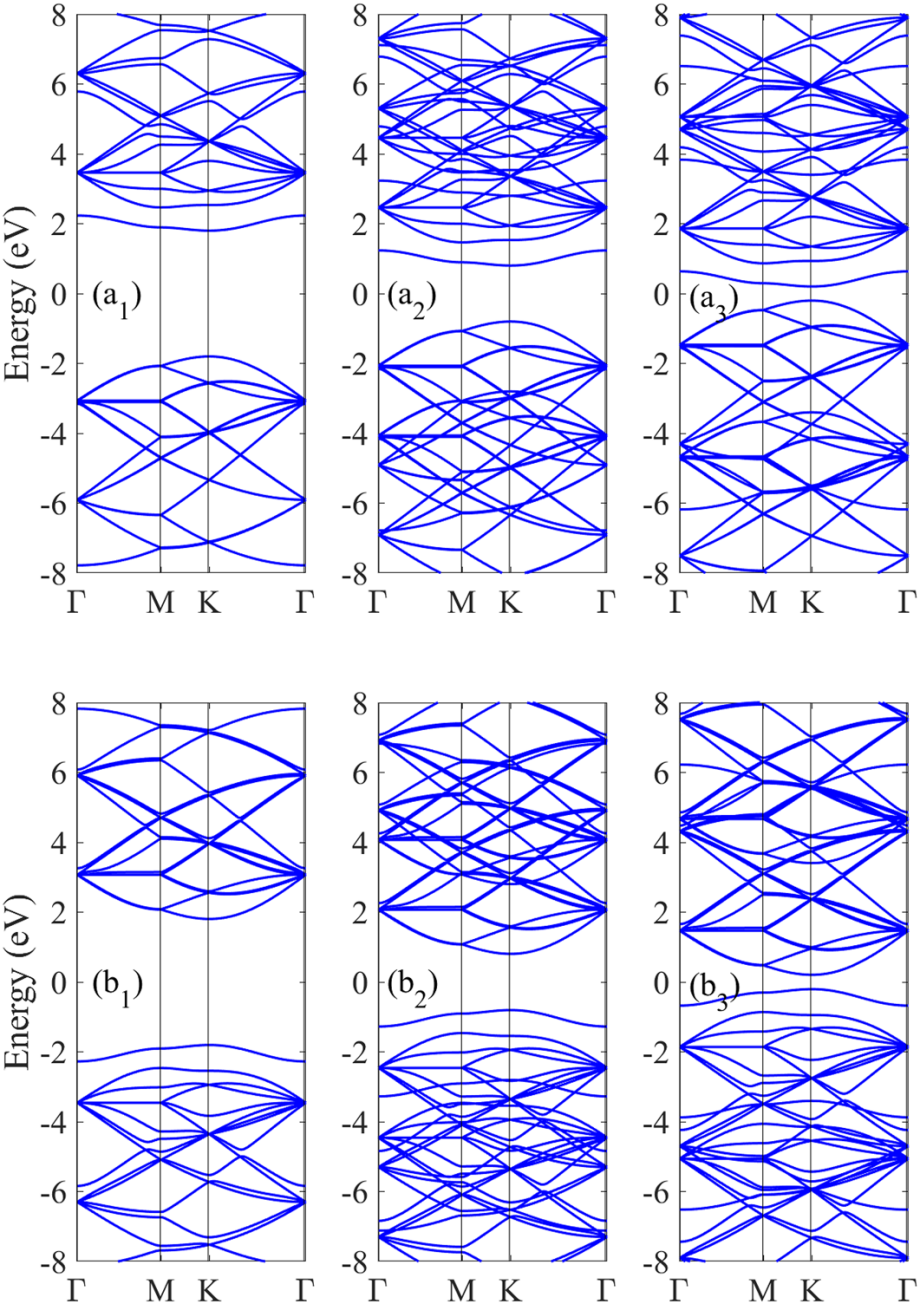
The tight binding results for the band structure of (**a1**–**a3**) n4-CB and (**b1**–**b3**) n4-CN doped h-BN structures in the presence of the magnetic fields Π = 0, 1, 2, respectively.

In this study, three types of doped h-BN structures have been selected for investigation: carbon dopant substitution on boron site [C_B_], carbon dopant substitution on nitrogen site [C_N_] and carbon dopants substitution on boron and nitrogen sites [C_BN_]. The electronic structure of doped h-BN with different impurity concentrations is shown in the Fig. 2 for N4 structure [supercell with four unit cells] with C_B_ and C_N_ impurity types. The electronic band structure was investigated in the symmetry path Γ–M–K–Γ.

A pure monolayer of BN is a wide band gap semiconductor. When it is doped with a carbon atom, a single subband is created within the band gap region. In the absence of a magnetic field (B_T_ = 0), N_4_-doped structure remains as direct band gap semiconductor and exhibits several valence and conduction asymmetry parabolic-like subbands on both sides of the Fermi level. For the C_B_ doped structure, the subband impurity above the Fermi level, shows flat dispersion in terms of wave vector k around the Γ point, especially in the MK direction (Fig. 2a1) and this feature is also occurs for the C_N_ doped structure. For the C_N_ doped structure, the impurity subband is located below the Fermi level in the valence band region and is flatness in terms of wave vector point around the K point (Fig. 2b1). In the case of C_B_-doped structures, the impurity subband above the Fermi level shows a flat dispersion in terms of wave vector k around the Dirac K point, especially in the MK direction (see Fig. 2a1). This feature is also observed in C_N_-doped structures and a similar flat impurity subband is present below the Fermi level around the K point in the valence band for C_N_ doping (Fig. 2b1). In the presence of a magnetic field, the Hamiltonian becomes spin-dependent, which leads to spin splitting of the subbands. Each valence and conduction subband splits into two separated bands, one for spin up and one for spin down. With increasing magnetic field strength, further modifications are induced, including the increasing of band edges and the merging and crossing of subbands. Another interesting and important feature is the band gap reduction of doped BN under magnetic field, attributed to the highest occupied molecular orbital (HOMO) and lowest unoccupied molecular orbital (LUMO) shifting closer together. This band gap reduction becomes more prominent with increasing magnetic field strength. These behaviors are not limited to h-BN, but have also been observed in other nanostructures, such as boron and nitrogen doped silicon carbon nanotubes and carbon nanotubes^68–70^.

**Figure 3:**
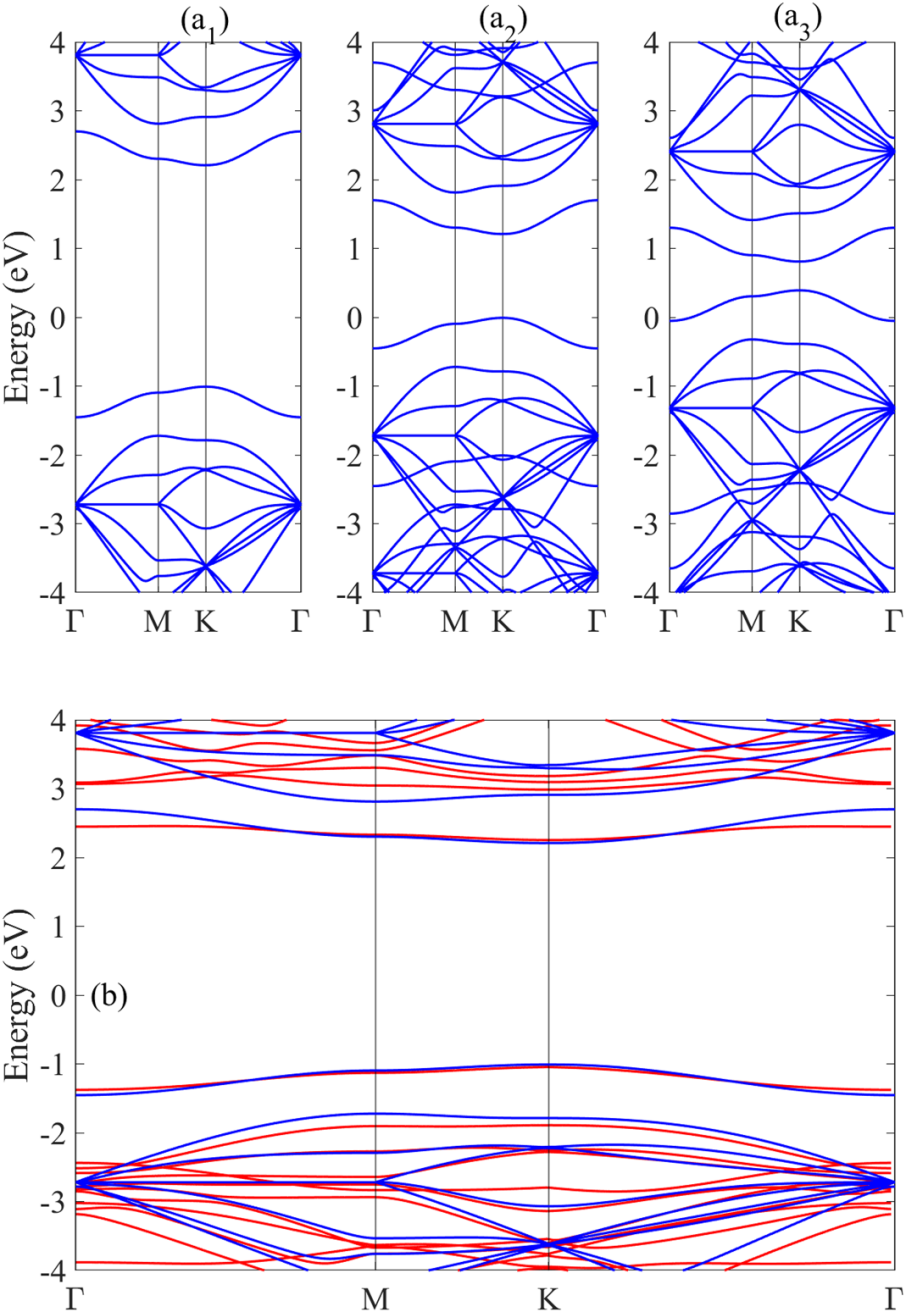
The tight binding results for the band structure of (**a1**–**a3**) n4-CBN doped h-BN structures in the presence of the magnetic fields Π = 0, 1, 2, respectively. (**b**) The tight binding (blue lines) and DFT (red lines) band structures. Good agreement is observed especially for the impurity subbands on both sides of the Fermi level, validating our tight binding approach.

To investigate the effects of double carbon doping on the electronic properties of h-BN with different unit cells, the band structure of the N_4_ structure with C_BN_ impurities is shown in Fig. 3. In the absence of a perpendicular electric field, this double-doped structure retains the direct band gap at the Dirac point like pristine BN. The two carbon impurities introduce separate flat bands in the valence and conduction bands (Fig. 3a1). Without a magnetic field, the band gap for C_BN_-doped structure is smaller than that the same structures with one carbon impurity. This demonstrates that the band gap size depends on both the type and concentration of dopants. The application and enhancement of a magnetic field induces a greater band gap reduction in N_4_-C_BN_ doped structures compared to those with just a single carbon impurity. Figure 3b shows the agreement between our tight binding results and DFT results for the band structure of CBN-doped h-BN. Good agreement is observed especially for the impurity subbands on both sides of the Fermi level, validating our tight binding approach.

**Figure 4:**
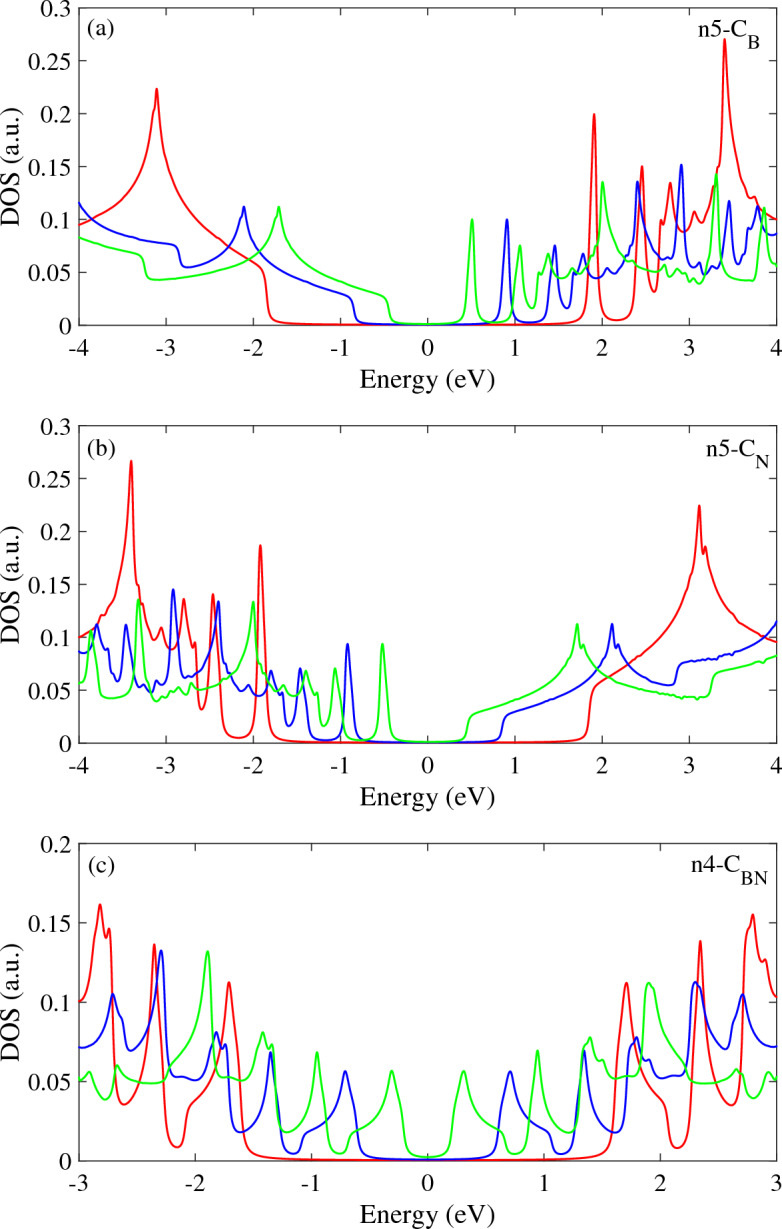
The DOS of the h-BN structure with (**a**) n5-CB, (**b**) n5-CN and (**c**) n4-CBN dopant types in the presence of the rescaled magnetic field $B_{T}$.

Figure 4 shows effects of the magnetic field on the DOS spectra for doped h-BN with different impurity types and concentration. Without the magnetic field, the wide band gap in the n_5_-C_B_ structure results in zero DOS intensity within |E|< 2 eV, because there are no available states for electrons in this energy range. The number of DOS peaks in the conduction region exceeds the valence region, attributable to the significant impact of the C dopant on the conduction bands near the Fermi level, regardless of magnetic field. The DOS spectrum for the n_5_-C_B_ structure exhibits significant distinct peaks with high intensity in energies around + 2 eV which arise from the dopant impurity sublevel in the band structure. When a magnetic field is applied, the first conduction peak moves toward the band gap region with decreasing intensity which leads to the band gap reduction in this structure. The DOS of n_5_-C_B_ structure, also has more and significant peaks in the positive energy region in the presence of a magnetic field. This is because the magnetic field enhances the effect of the dopant impurity sublevel on the conduction bands. In contrast, the DOS in the negative energy region has a single peak, which is due to the absence of the dopant impurity sublevel in this region.

Figure 4b presents the influence of an applied magnetic field on the DOS for h-BN doped with C_N_ impurity types. In the absence of a field, negligible DOS intensity is observed across a wide energy range around the Fermi level. For the C_N_ dopant type, more DOS peaks appears in the valence region, compared to the conduction region. This asymmetry in C_N_ doped structure, arises due to the significant influence of the carbon dopant on the valence band edges. For n_5_-C_N_, distinct high-intensity DOS peak appears around − 2 eV originating from the dopant impurity subband. Under an applied magnetic field, this impurity peak shifts toward the Fermi level, which leads to reduction of the magnetic field-induced band gap. As depicted in Fig. 4c, the presence of dual carbon dopants introduces two distinct peaks on either side of the Fermi level. This arises as the two dopants substantially influence both the valence and conduction bands. Under an applied magnetic field, the two first peaks nearest to the Fermi level, shift closer together and this leads to the band gap reduction.

It can be concluded that the number, position and intensity of the peaks strongly modified with dopant type and concentration. Also, the magnetic field creates many additional peaks inside the band gap region by splitting the valence and conduction subbands and moving them toward the Fermi level.

### Thermal properties

Thermal properties of materials are affected by electronic and phononic contributions. This study focuses on the electronic contribution under conditions of high electron concentration and short phonon mean free path and the phononic contribution is not considered here^71^.

**Figure 5:**
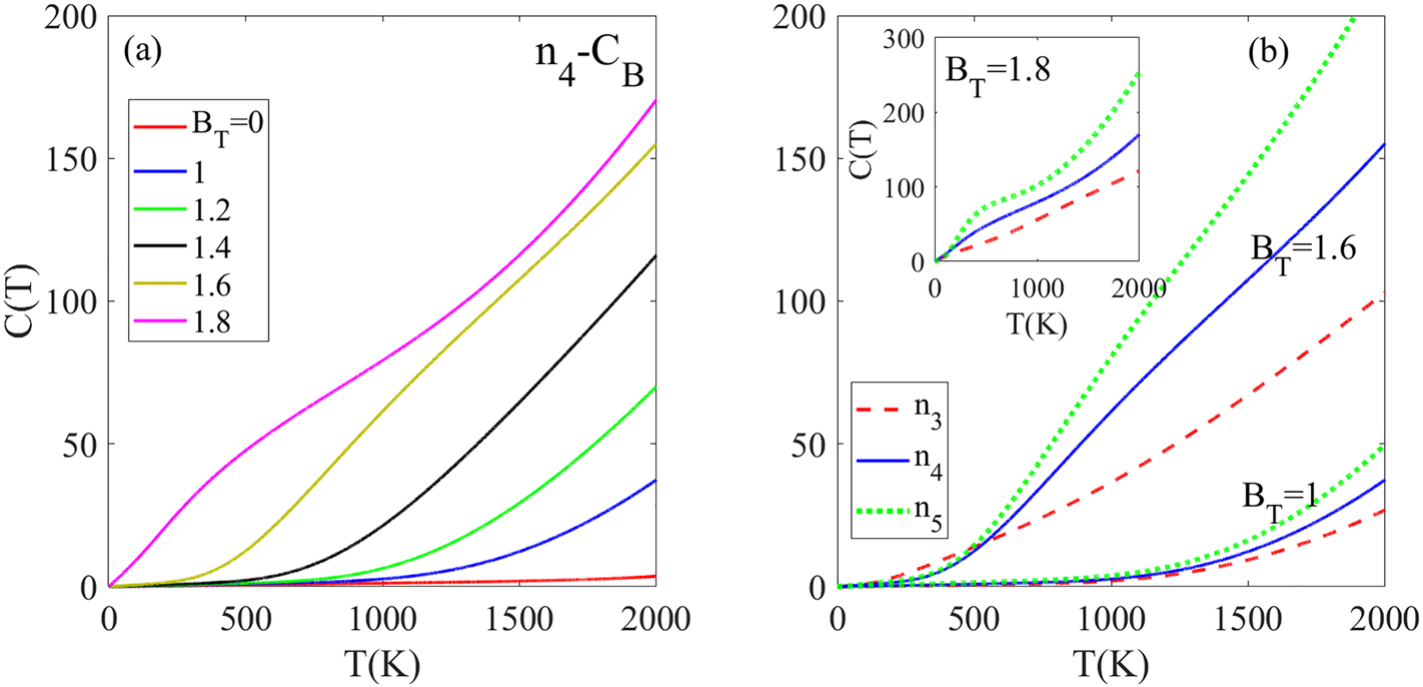
(**a**) Temperature dependence of heat capacity [in an arbitrary unit] for n4-CB doped h-BN monolayer at different applied rescaled magnetic field strengths. (**b**) Heat capacity as a function of temperature for CB-doped h-BN with n3, n4, and n5 dopant concentrations under rescaled magnetic fields of 1, 1.6, and 1.8.

The temperature dependence of the heat capacity of h-BN with the carbon impurity and magnetic field has been investigated using the Eq. 13. For this purpose, the n4-doped structure with C_B_, C_N_ and C_BN_ dopant types are selected. In the T < 2000 K, the $C\left( {T,B_{T} = 0} \right)$ for n4-CB doped structure exhibits near to zero intensity (red line in Fig. 5a) due to its wide band gap which acts as a barrier potential for the charge transition. Below 2000 K, the charge carriers lack sufficient thermal energy for transfer to higher levels. The magnetic field decreases the band gap and significantly alters the DOS peak intensities near the Fermi level. Due to these modifications, the density of charge carrier increases and with lower thermal energy, more charge carriers can be transfer to the higher levels. So, in non-zero magnetic field B_T_ ≠ 0, the heat capacity of n_4_-C_B_ case becomes non zero below 2000 K. As shown in the Fig. 5a, the $C\left( {T,B_{T} = 1} \right)$ remains zero below 1000 K and becomes non zero above this temperature region. The increasing rate of heat capacity with temperature depends on the magnetic field strength. At higher fields of B_T_ = 1.4 and 1.6, the heat capacity becomes non zero and rises when temperature exceeds 500 K and 300 K, respectively. Additionally, at B_T_ = 1.8, C is nonzero across all temperatures below 2000 K and grows linearly with increasing temperature.

Figure 5b compares the effects of dopant impurity on the heat capacity C(T) for different doped structures (n_3_, n_4_, n_5_ supercells) under a constant magnetic field. At B_T_ = 1, all structures exhibit identical C(T) intensity below 1500 K. Above 1500 K, n_5_ has the largest C(T) while n3 has the smallest. This trend also occurs at higher magnetic fields of B_T_ = 1.6 and 1.8, where n_5_ and n_3_ display the largest and smallest C(T) respectively for T > 500 K. Notably, the increasing rate of C(T) with temperature is enhanced by increasing magnetic field. Furthermore, at higher fields, the difference in C(T) between structures increases. In summary, besides dependence on magnetic field, C(T) also strongly depends on the impurity concentration, with lower concentrations exhibiting larger C(T).

**Figure 6:**
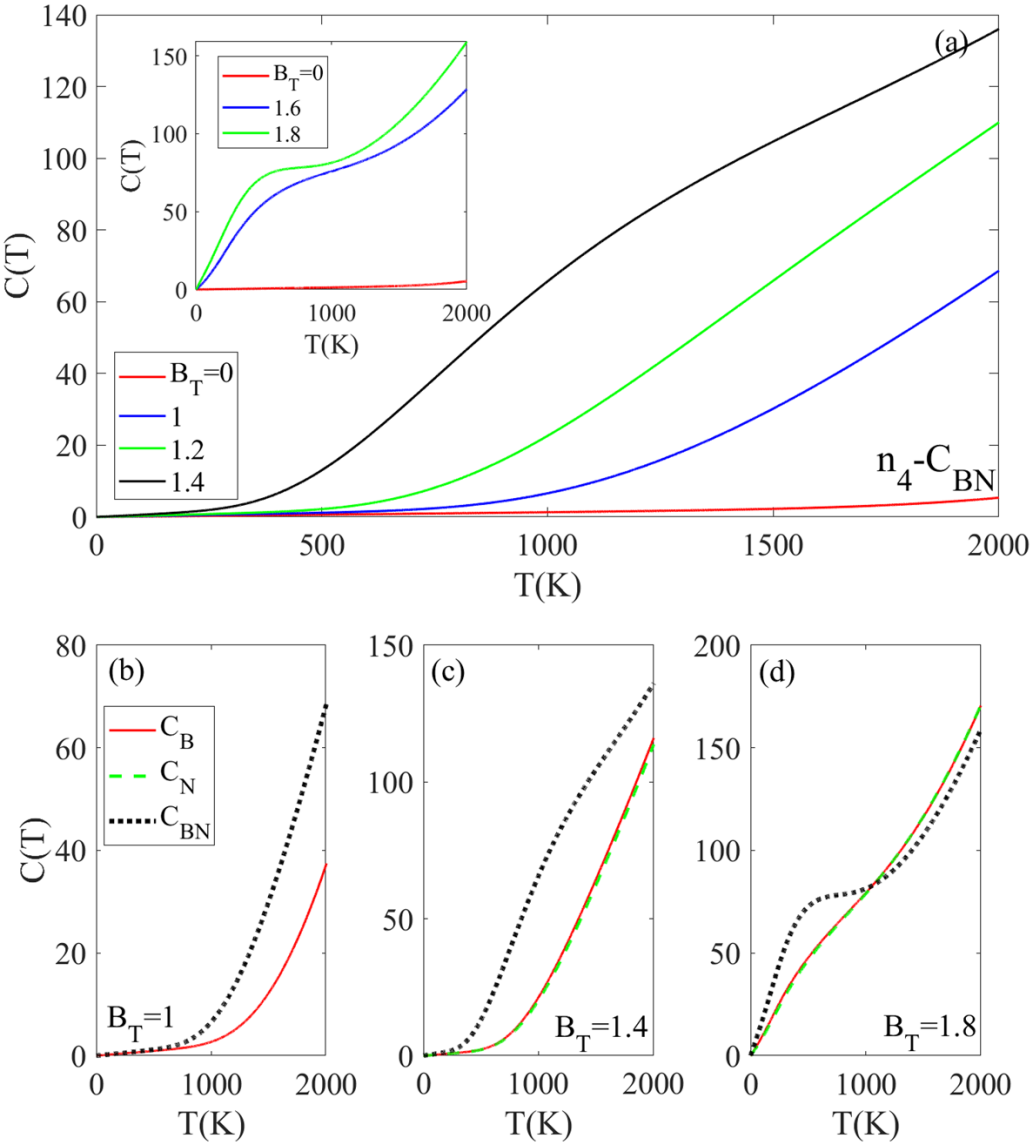
(**a**) Heat capacity [in an arbitrary unit] as a function of temperature for n4-CBN doped h-BN monolayer at various applied rescaled magnetic fields. (**b**–**d**) Comparison of heat capacity versus temperature for n4 doping concentration with CB, CN, and CBN dopant types under rescaled magnetic fields of 1, 1.4, and 1.8, respectively. The results demonstrate tunable heat capacity through the magnetic field, dependent on both dopant type and concentration.

The temperature dependence of the specific C(T) for the n_4_-C_BN_ doped structure containing two carbon impurity atoms is presented in Fig. 6a. In the absence of an external B_T_, the C(T) intensity remains close to zero below 2000 K. This is attributed to the wide band gap of the n_4_-C_BN_ doped structure, which inhibits thermal excitation of charge carriers to higher energy levels. The C(T) as a function of temperature for the n_4_-C_BN_ doped structure exhibits a strong dependence on the applied external magnetic field B_T_. In B_T_ = 0, C(T) remains negligible up to 2000 K due to the wide band gap of the n_4_-C_BN_ which prevents significant thermal excitation of charge carriers. However, the application of a magnetic field induces a reduction in the band gap via the Zeeman Effect, thereby decreasing the energy required for charge carriers to be excited across the band gap. At B_T_ = 1, the C(T) remains negligible until approximately 700 K, above which it becomes nonzero. When the BT is increased to 1.4, this threshold temperature rapidly rising decreases drastically to around 280 K. With stronger B_T_ = 1.6 and 1.8, the low C(T) region disappears and at these field strengths, C(T) starts increasing rapidly above 0 K. Figure 6b–d compare the effects of different dopant types (C_N_, C_B_, C_BN_) on the C(T) in the presence of varying magnetic field strengths. At lower field strengths of B_T_ ≤ 1.4, the C(T) values for the C_N_ and C_B_ doped structures are approximately equal to each other, and both are lower than the C(T) for the C_BN_ doped structure. However, at the higher field strength of B_T_ = 1.8, the differences in C(T) between the dopant types decrease as the temperature rises above 1000 K (Fig. 6d).

**Figure 7:**
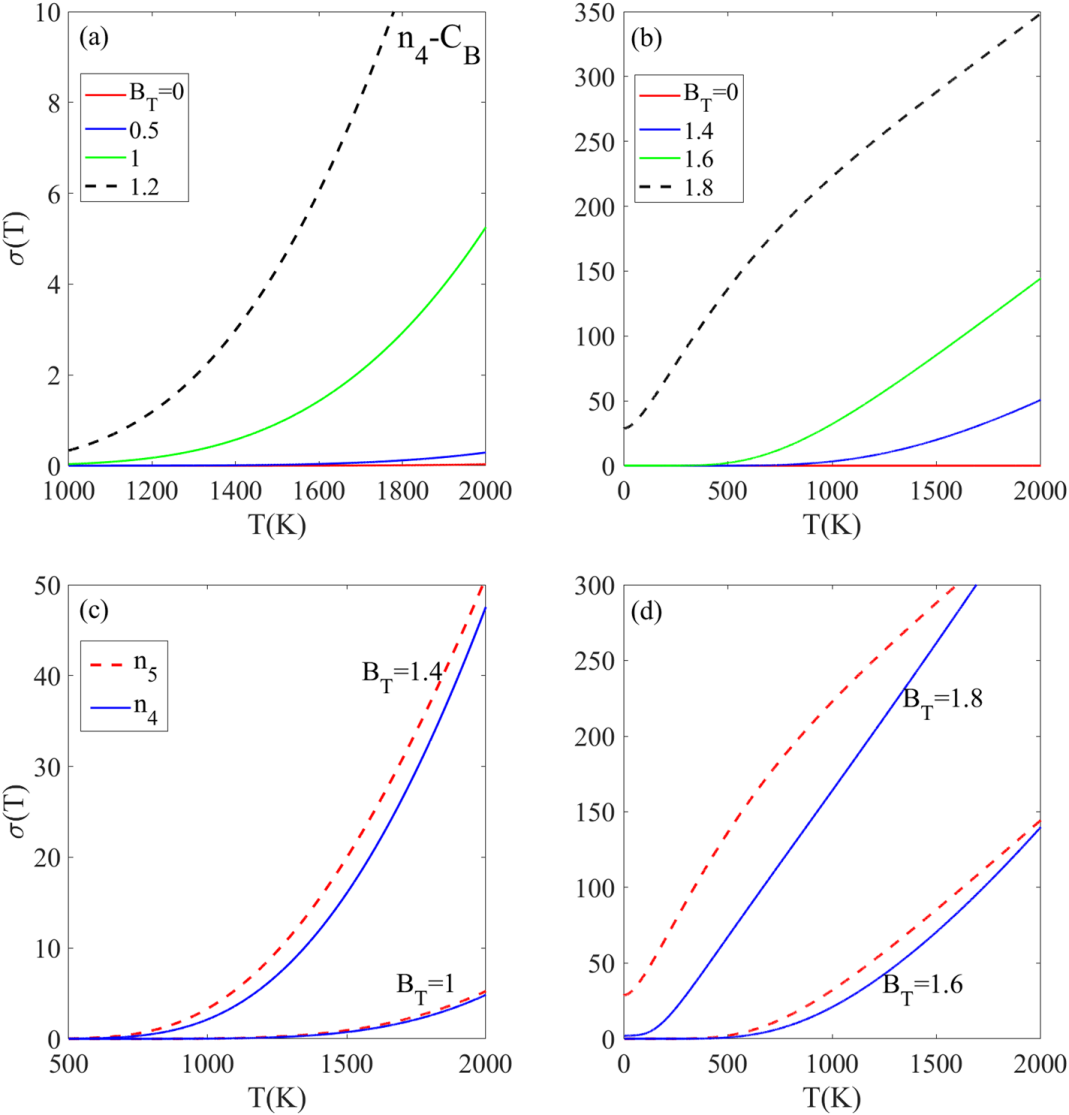
(**a**, **b**) Temperature dependence of electrical conductivity [in an arbitrary unit] for n4 CB-doped h-BN monolayer under different applied rescaled magnetic field strengths. (**c**, **d**) Comparison of electrical conductivity as a function of temperature for n4 and n5 CB doped h-BN at various rescaled magnetic field strengths. The results demonstrate tunable electrical conductivity through the magnetic field, dependent on both dopant concentration and magnetic field intensity.

Figure 7 shows the electronic contribution to electrical conductivity σ(T) versus temperature for C_B_-doped structures with different doping concentrations and magnetic fields. The temperature dependence of the σ(T) depends on different parameters such as the band gap of doped structure, density of excited charge carriers and their mobility. Without an external B_T_, the wide band gap about 3.6 eV for the n_4_-C_B_ doped structure prevents charge carrier excitation, giving zero σ(T) below 2000 K as seen in Fig. 7a. The application of an external magnetic field causes Zeeman splitting of the electronic energy levels in the doped h-BN, which reduces the band gap. The band gap decreases to 0.76 eV and 0.32 eV at magnetic field strengths of B_T_ = 1.4 and 1.6, respectively. The band gap then vanishes completely at a magnetic field of BT = 1.8. The smaller band gap enables increased thermal excitation of electrons from the valence band to the conduction band at lower temperatures. The greater number of thermally excited charge carriers leads to an increase in σ(T). Consequently, compared to the absence of the magnetic field, the σ(T) becomes nonzero and begins to rise at temperatures above 1000 K for magnetic fields of B_T_ ≥ 1.4, because above 1000 K, the increasing temperature provides sufficient thermal energy to excite a more electrons across the reduced band gap. Notably at T = 1000 K, the conductivity σ(T) remains negligible for B_T_ = 1.4 compared to B_T_ = 1.6, due to the smaller reduction of the band gap. Note that, at lower magnetic fields of B_T_ ≤ 1.4, the reduction in band gap is not enough to allow for significant thermal excitation of carriers below 1000 K and as a result, the σ(T) stays negligible or zero. With increasing applied magnetic fields of B_T_ ≥ 1.6, the σ(T) becomes nonzero above 500 K, respectively. This is because the band gap reduction caused by this magnetic field strength is still large enough to prevent thermal excitation of carriers below 500 K. However, at the higher magnetic field of B_T_ = 1.8, the band gap has been sufficiently reduced so that the σ(T) reaches its maximum intensity and becomes nonzero at temperatures above 0 K. It can be concluded that the C_B_ doped structure exhibits a larger band gap when is influenced with weaker BT, so, its σ(T) increases when a stronger B_T_ is applied.

Figure 7c and d show the σ(T) for C_B_-doped h-BN structures with different impurity concentrations n_4_ and n_5_, under various magnetic fields. At B_T_ = 1, the σ(T) for both structures is zero below 1200 K and approximately equal. When the magnetic field is increased to B_T_ = 1.4, the σ(T) remains zero below 500 K but significantly increases above this temperature, with a higher intensity for the n_5_ doped structure. This increase in electrical conductivity is due to the magnetic field’s effect on the band gap, as the gap decreases with increasing B_T_. Note that, the rate of band gap reduction with respect to B_T_ is greater for the n_5_ structure compared to the n_4_ structure. At stronger magnetic fields of B_T_ = 1.6 and 1.8, the difference between σ(T) of n_4_ and n_5_ increases, with n_4_ having a lower σ(T) intensity. This is due to n4 having a larger band gap than n5 at these field strengths. These findings also can be explained by the impurity concentration. The doped h-BN structure with lower impurity concentration (n_5_) allows electrons and holes to move more freely with less scattering. This enables more efficient heat conduction and leads to higher electrical conductivity, when compared to the structure with higher impurity concentration (n_4_) which causes more scattering.

**Figure 8:**
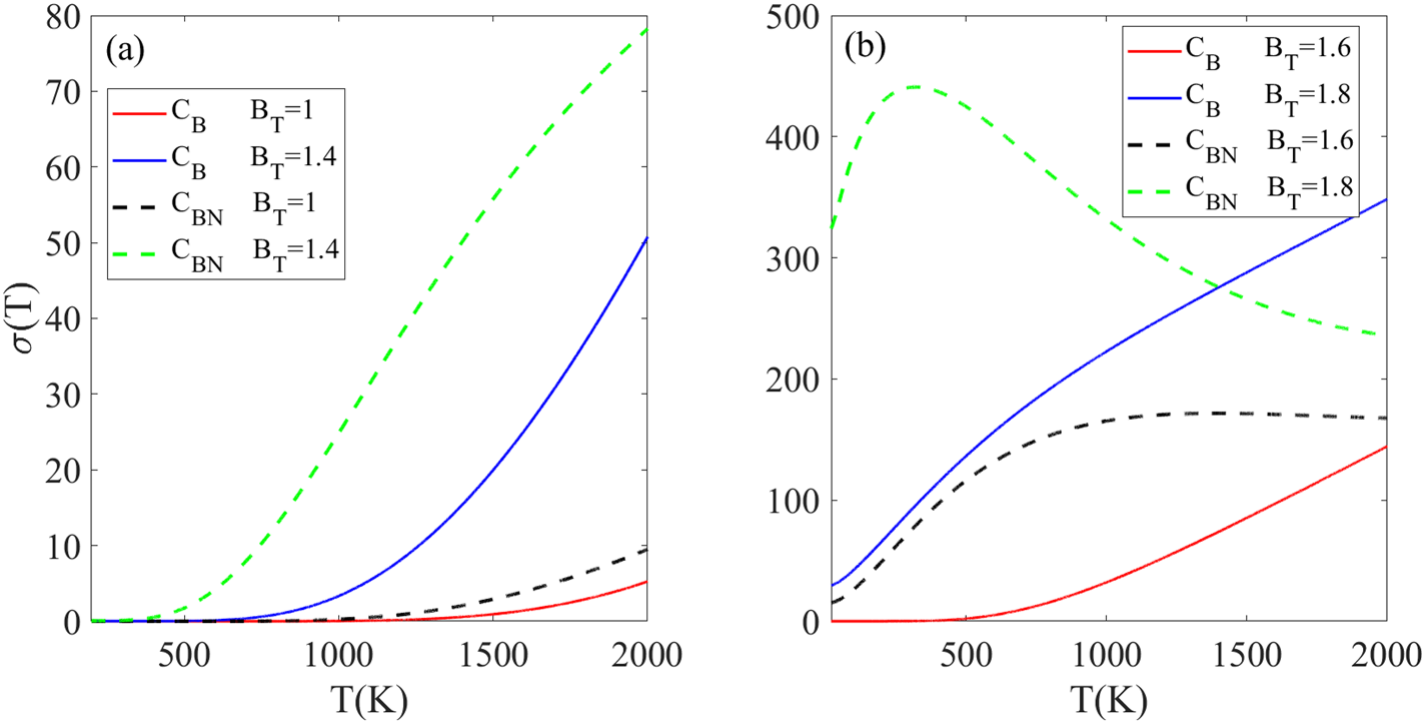
Comparison of electrical conductivity [in an arbitrary unit] versus temperature for n4 doping concentration with CB and CBN dopant types under different rescaled magnetic fields.

The results in Fig. 8 indicate that the electrical conductivity of doped h-BN exhibits a strong dependence on several external factors, namely temperature, dopant type and concentration, and magnitude of the applied magnetic field. By engineering these external factors, significant changes can be induced in the electrical conductivity. The tunable electrical conductivity of doped h-BN originates from the changes in the electronic band structure caused by the substitution of carbon atoms in h-BN with boron or nitrogen dopants. The impurity atoms introduce new energy levels within the band gap that are sensitive to both doping concentration and applied magnetic field strength. Tuning the external factors leads to changes in the band structure, specifically decreasing the size of the band gap. The modifications become more pronounced as the magnetic field increases. Consequently, the narrowed band gap requires less energy for thermal excitation of charge carriers across the gap, resulting in higher electrical conductivity. For all selected doped structures, the electrical conductivity is zero in the finite temperature region indicated by TZ. The TZ region depends on the impurity concentration and magnetic field strength. At a magnetic field of B_T_ = 1.4, the σ(T) remains zero below temperatures of T_Z_ = 700 K and 400 K for the C_B_ and C_BN_ doped structures, respectively (Fig. 8a). Above these temperatures, the σ(T) increases from zero for both doped structures as the temperature further increases because the higher temperatures provide the required thermal energy to excite a greater number of charge carriers to higher energy levels. At B_T_ = 1, the σ(T) of the n_4_-C_B_ doped structure is smaller than the n_4_-C_BN_ doped structure, and this relationship is unchanged as B_T_ increases to 1.4. This can be explained by the smaller band gap in the C_BN_-doped structure compared to the C_B_-doped structure, which requires less thermal energy to excite charge carriers to higher levels. Figure 8b shows the behavior of σ(T) in the presence of stronger magnetic fields B_T_ = 1.6 and 1.8, for the C_B_ and C_BN_ doped structures with the same n_4_ supercell. At B_T_ = 1.6, the σ(T) of the CB-doped structure is zero below T_z_ < 500 K [due to the non-zero band gap] and increases above 500 K. This differs from the behavior of the C_BN_-doped structure at the same magnetic field of B_T_ = 1.6, which has a non-zero σ(T) even below 2000 K. This difference can be attributed to the disappearance of the band gap in the n_4_-C_BN_ structure when B_T_ = 1.6. However, the behavior of both structures at B_T_ = 1.8 reveals an interesting difference. While the σ(T) is non-zero below 2000 K for both, the C_B_ structure displays a linearly increasing σ(T) pattern, whereas the C_BN_ structure exhibits a decreasing σ(T) above 500 K. The decreasing σ(T) pattern for C_BN_ at the high magnetic field of B_T_ = 1.8 can be explained by scattering among charge carriers. The C_BN_ band gap becomes very narrow at B_T_ = 1.8. As temperature increases, more charge carriers gain enough energy to occupy higher energy levels, which rapidly increases the population of excited carriers. This leads to increased scattering interactions between charge carriers at higher energy levels as temperature increases. These more scattering interactions between carriers lead to the reduced σ(T) exhibited by the C_BN_-doped structure. It can be concluded that (i) C_BN_ doping gives higher conductivity than C_B_ doping due to a smaller band gap and (ii) high densities of scattered carriers can decrease conductivity as seen for C_BN_-doped h-B_N_ at high magnetic fields.

**Figure 9:**
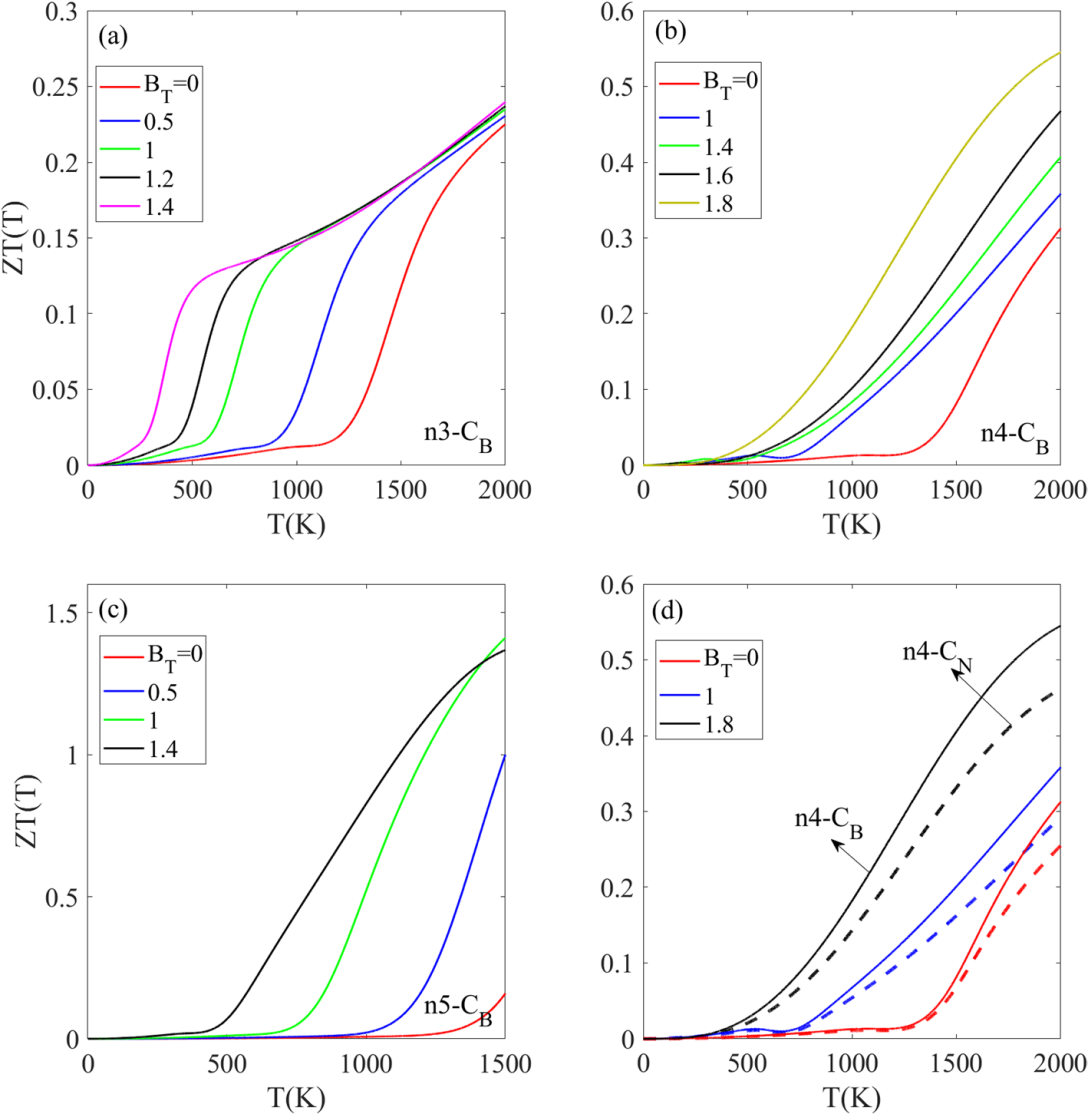
Tuning thermoelectric figure of merit ZT(T) in carbon doped h-BN through magnetic fields. (**a**–**c**) Temperature-dependent ZT(T) for CB doped h-BN monolayers with n3, n4, and n5 dopant concentrations under applied rescaled magnetic fields. (**d**) Comparison of ZT(T) as a function of temperature for n4 doped h-BN with CB and CN dopant types at various rescaled magnetic field strengths.

The temperature behavior of the electronic figure of merit ZT(T) dependent on dopant type and concentration is investigated in Fig. 9 for the N_3_, N_4_, and N5 doped structures with C_B_ impurity. In the absence of a magnetic field (red lines in Fig. 9), ZT(T) has negligible values below approximately 1000–1200 K, then sharply increases above this temperature range. This pattern occurs independently of impurity concentration for all selected cases. Also, similar temperature-dependent increases in ZT(T) with temperature have also been reported for Silicene and Germanene^72,73^ and MoTe2^74^. Applying a magnetic field to each structure causes the ZT(T) increase to begin at lower temperatures. For example, for the n_3_-C_B_ doped structure, the ZT(T) becomes non zero and increases above 500 K and 260 K when magnetic field becomes B_T_ = 1 and 1.4, respectively. The magnetic field B_T_ has another significant effect on ZT(T) intensity, creating larger ZT(T) values at higher B_T_ strengths relative to weaker B_T_. As the temperature increases to 2000 K, each chosen structure displays a unique ZT(T) behavior. As temperature increases towards 2000 K, the ZT(T) for n_3_-C_B_ converges to the same value and the difference in ZT(T) for varying magnetic field strengths decreases. For the n_4_-C_B_ doped structure, the difference in ZT(T) between varying magnetic field strengths increases slightly, with stronger B_T_ exhibiting higher ZT(T) intensity (Fig. 9b). In the presence of non-zero magnetic fields, the ZT(T) of the n_5_-C_B_ doped structure displays a peak at temperature T_M_ (Fig. 9c) and As B_T_ increases, this peak position in the n_5_-C_B_ ZT(T) shifts to lower temperatures. Notably, comparison of the ZT(T) intensity for the n_3_, n_4_, and n_5_ doped structures with the same impurity type shows that n5 exhibits the highest intensity compared to the other cases. Based on the above results, it can be concluded that the ZT(T) is higher for structures with lower impurity concentrations and increases as the impurity concentration is reduced. Figure 9d shows the effects of the dopant type on temperature dependence of ZT(T) for n_4_-C_B_ and n_4_-C_N_ doping at various magnetic fields. The results demonstrate that at a given magnetic field strength, the C_B_ doped structure exhibits a higher ZT(T) compared to the C_N_ doped structure.

**Figure 10:**
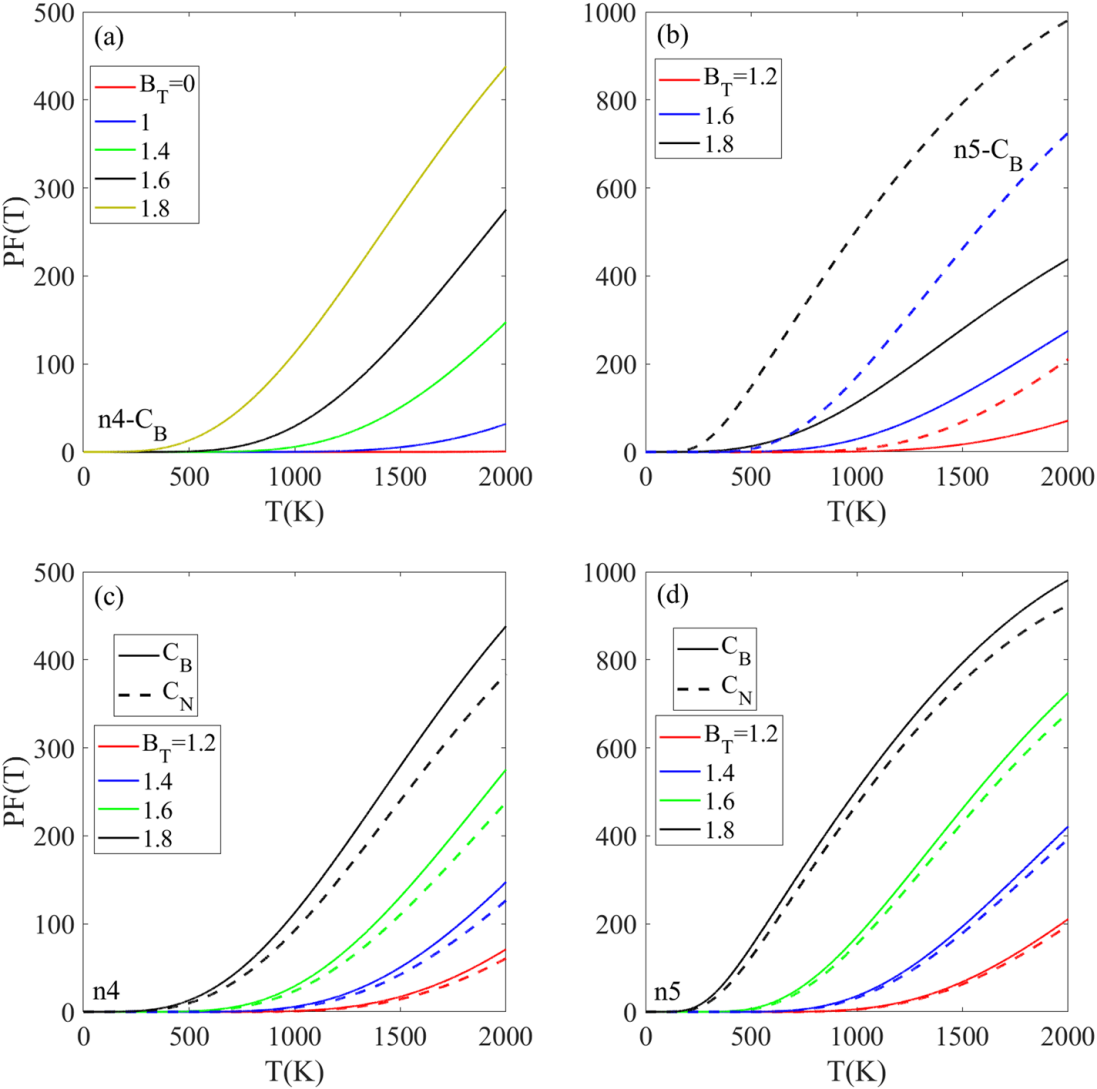
(**a**, **b**) Temperature-dependent of power factor PF(T) [in an arbitrary unit] for CB doped h-BN monolayers with n4, and n5 dopant concentrations under applied rescaled magnetic fields. (**c**, **d**) Comparison of PF(T) as a function of temperature for n4 doped h-BN with CB and CN dopant types at various rescaled magnetic field strengths.

Figure 10 displays the influences of magnetic field, impurity type, and concentration on the temperature-dependent power factor PF(T) for the chosen structures. For the N_4_ structure with C_B_-type doping, the power factor PF(BT = 0) remains near zero below 2000 K. When the magnetic field B_T_ = 1 is applied, the PF(T) rises above 1500 K, and at B_T_ = 1.8 the increasing rate is substantially enhanced with temperature (Fig. 10a). Figure 10b compares the PF(T) for n_4_ and n_5_ doped structures with C_B_ impurities at varying B_T_. At B_T_ = 1.2, the PF(T) is zero below 1000 K and rises above 1000 K for both structures, with greater intensity for the n5 structure. As magnetic field increases to B_T_ = 1.6, the PF(T) for n_5_ grows at a faster rate compared to the n_4_ structure and their differences more increased as temperature reaches 2000 K. The results indicate that reducing the impurity concentration has a greater impact on PF(T) compared to increasing the magnetic field strength B_T_, as evidenced by Fig. 10b, where the PF(T) at BT = 1.6 for n_5_-C_B_ doping exceeds the PF(T) at B_T_ = 1.8 for n_4_-C_B_ doping. In summary, for a given impurity type, the PF(T) can be increased by both raising the magnetic field strength and lowering the impurity concentration. However, reducing the impurity level has a greater enhancing effect on PF(T) compared to increasing the magnetic field. As shown in the Fig. 10c and d, the intensity of PF(T) with the C_B_ impurity type is larger than that corresponding doped structure with the C_N_ impurity type and this behavior is independent of the magnetic field strength and impurity concentration.

## Conclusion and outlooks

Optimizing the thermoelectric performance of carbon-doped h-BN monolayers through tuning the doping concentration, magnetic field strength, and impurity type is investigated through tight binding model, using Green’s function approach and Kubo formalism. Through determination of accurate tight-binding parameters, excellent agreement was obtained between the electronic properties of the tight-binding model and corresponding Density Functional Theory (DFT) calculations for doped h-BN structures across a range of impurity types and concentrations. The effects of external factors such as carbon doping type and concentration as well as applied magnetic field strength were systematically studied through their influence on key thermoelectric properties including the electronic density of states, electrical conductivity, heat capacity, electronic figure of merit, and power factor. The electronic band structure and density of states of monolayer hexagonal boron nitride (h-BN) are significantly modified by carbon doping. Carbon dopants induce subband splitting and merging in the electronic structure, altering the number, position, and magnitude of peaks in the density of states. Under an applied magnetic field, additional peaks appear in the band gap region of C-doped h-BN, originating from sublevel splitting which leads to a reduction of the band gap in carbon-doped h-BN.

These modifications to the electronic structure directly influence the electrical conductance and thermoelectric properties of h-BN as follows:

- Double carbon doping improves electrical conductivity compared to single doping, but strong carrier scattering reduces it at high magnetic fields.
- Heat capacity varies markedly with dopant concentration, displaying higher values at lower dopant levels.
- At a given magnetic field, the CB doped structure exhibits a higher electronic figure of merit ZT(T) than the CN doped structure, with ZT(T) increasing as impurity concentration decreases.
- For a given dopant type, the power factor PF(T) can be raised by increasing magnetic field and lowering impurity concentration.

The ability to tune the electronic structure and band gap of h-BN through carbon doping and applied magnetic fields opens up new possibilities for engineered optical and electronic properties. This work shows routes to enhance h-BN for advanced optoelectronic and power generation applications using band gap modification through doping combined with electronic structure changes induced by magnetic field.

### Supplementary Information

Supplementary Information.

## Data Availability

The datasets used and analyzed during the current study available from the corresponding author on reasonable request.
