# Supplementary material for: Optimizing thermoelectric performance of carbon-doped h-BN monolayers through tuning carrier concentrations and magnetic field
Source: Sci Rep. 2023 Nov 10;13:19623. doi: 10.1038/s41598-023-46116-w (PMC10638448; doi:10.1038/s41598-023-46116-w)
Supplement: Supplementary file 1 — Supplementary Information. [file 41598_2023_46116_MOESM1_ESM.docx]

**Supporting File**

**Optimizing Thermoelectric Performance of Carbon-Doped h-BN Monolayers through Tuning Carrier Concentrations and Magnetic Field**

**Somayeh Behzad, Raad Chegel^2^**

**^1^Department of Engineering Physics, Kermanshah University of Technology, Kermanshah, Iran**

**^2^Department of Physics, Faculty of Science, Malayer University, Malayer, Iran**

**DFT calculations:**

The Figure S1 shows the DFT calculation for the electronic band structure of the doped SiC with different impurity types and impurity concentrations. In this study, the DFT calculations are performed using the Spanish initiative for electronic simulations with thousands of atoms (SIESTA) package [1]. All the calculations were carried out with a the double-zeta plus polarization atomic orbital (DPZ) basis set and for the exchange and correlation terms, the Local Density Approximation (LDA) according to the Ceperly and Alder (CA) parameterization is used [2]. The cutoff energy for the plane waves is set to 420 Ry and all atoms were fully relaxed until the residual forces were smaller than 0.02 eV/Å. A minimum vacuum distance of 20 Å between neighboring images is used to avoid the interaction between the adjacent layers. The Brillouin zone integration is performed by using the Gamma-centered Monkhorst-Pack scheme with 12 × 12 × 1 k-points.

**Finding the required tight binding parameters:**

The tight binding model Hamiltonian requires parameters including nearest neighbor hopping integrals and on-site atomic energies. These parameters can be determined by fitting the tight binding band structure to match density functional theory (DFT) band structures. In this work, we obtained the tight binding parameters by fitting to DFT band structures for various impurity types and concentrations. Figure S1 shows the tight binding (blue) and DFT (red) band structures for n3 and n4 doped h-BN, exhibiting excellent agreement in the band gap. Through this fitting procedure, we obtained a parameterized tight binding model that accurately captures the band structure of carbon-doped h-BN based on first-principles DFT calculations.


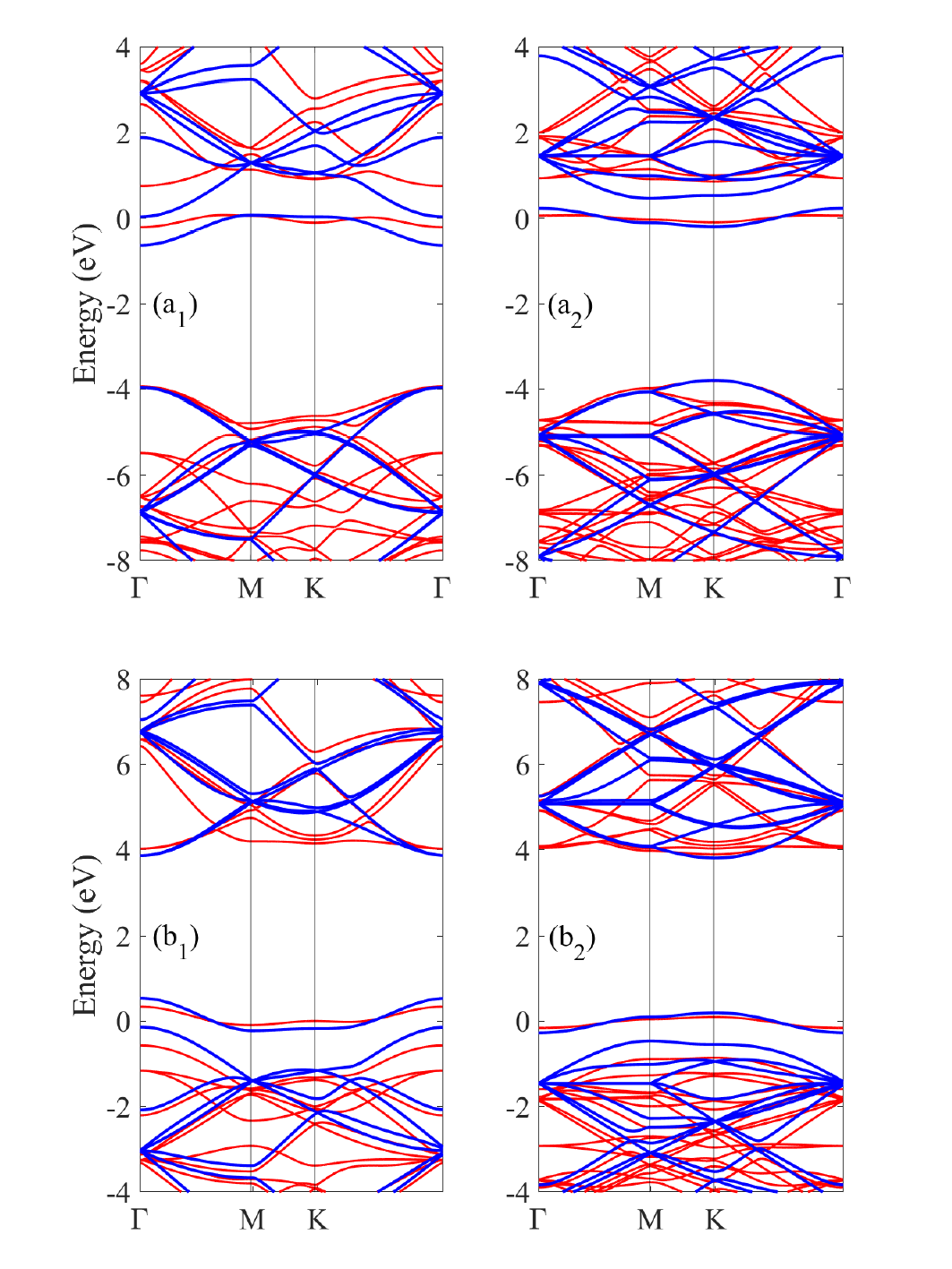


**Figure S1.** The DFT (red lines) and tight binding (blue lines) results for the electronic structure of (a_1_)-(a_2_) CB and (b_1_)-(b_2_) CN doped structures with n_3_-n_4_ unit cells, respectively.

**Calculation the thermoelectric based on the Kubo formula:**

Using a tight binding model Hamiltonian, we investigated the effects of electric field, magnetic field, and impurity concentration on the temperature dependence of thermoelectric properties in carbon-doped h-BN.

**Obtaining band structure:**

1. The real-space Hamiltonian matrix is constructed [Eq. 1] for the system. Different impurity concentrations are modeled by increasing the number of unit cells.
2. The k-space Hamiltonian is obtained through Fourier transformation of the fermion creation and annihilation operators.
3. Under an applied magnetic field Π, the Hamiltonian becomes spin-dependent, represented as a $N^{2}$×$N^{2}$ matrix.
4. The electronic band structure $E(\boldsymbol{k})$ can be obtained by solving the Schrödinger equation.

**Density of States (DOS)**

1. The electron equation of motion [$\sum_{s} \left[ \left( E\boldsymbol{I}-\boldsymbol{\Omega}(B_{T}) \right)\delta_{is}+\boldsymbol{t}_{is} \right]\boldsymbol{G}_{sj}\left( E \right)=\boldsymbol{I}\delta_{ij}$] is used to derive the Green's function matrix [Eq. 3].
2. Fourier transformation of the Green's function to k-space allows calculation of the density of states from the imaginary part of the Green's function [$-{\frac{\mathbf{1}}{\boldsymbol{\pi}}\boldsymbol{Im}[\boldsymbol{G}}_{jj}\left( E \right)]$.
3. Based on the DOS spectra, the heat capacity can be calculated. [$C(T)=\int_{-\infty}^{\infty} \varepsilon\left[ \frac{\partial f\left( \varepsilon,T \right)}{\partial T} \right]D\left( \varepsilon\right) d\varepsilon$]
4. The spectral function $\boldsymbol{A}\left( \boldsymbol{k},\varepsilon\right)=-2Im\hat{G}\left( k,\varepsilon\right)$, related to the density of states, is required for computing the thermoelectric properties.

**Thermoelectric properties**

1. According to the spectral function of the Green’s function, the transport coefficients [$\Upsilon_{qq^{'}}$] is given by the Kubo relation, as:

$$\Upsilon_{11}\left( T,\Pi\right)=\frac{1}{\beta} \int_{-\infty}^{+\infty} \left[ \frac{\partial f\left( \varepsilon\right)}{\partial\varepsilon} \right]\frac{d\varepsilon}{2\pi} \sum_{k,p} \left( \nu_{k}^{\left( p \right)}A_{p}\left( k,\varepsilon\right) \right)^{2}$$

$$\Upsilon_{12}\left( T,\Pi\right)=\frac{1}{\beta} \int_{-\infty}^{+\infty} \left[ \frac{\partial f\left( \varepsilon\right)}{\partial\varepsilon} \right]\frac{d\varepsilon}{2\pi} \sum_{k,p} \left( E_{p}\left( \boldsymbol{k},B_{T} \right) \right)^{1}\left( \nu_{k}^{\left( p \right)}A_{p}\left( k,\varepsilon\right) \right)^{2}$$

$$\Upsilon_{22}\left( T,\Pi\right)=\frac{1}{\beta} \int_{-\infty}^{+\infty} \left[ \frac{\partial f\left( \varepsilon\right)}{\partial\varepsilon} \right]\frac{d\varepsilon}{2\pi} \sum_{k,p} \left( E_{p}\left( \boldsymbol{k},B_{T} \right) \right)^{2}\left( \nu_{k}^{\left( p \right)}A_{p}\left( k,\varepsilon\right) \right)^{2}$$

2. Based on the transport coefficients, the electrical conductivity is proportional to $\Upsilon_{11}\left( T,\Pi\right)$.
3. The power factor is directly related to the electrical conductivity and it is defined as PF= σS^2^ where σ and S are the electrical conductivity and seebeck coefficient, respectively. Based on the transport coefficients, the power factor can be defined as:

$$\mathrm{PF}\left( T \right)\equiv\frac{[\Upsilon_{12}]^{2}}{T^{2}\Upsilon_{11}}$$

4. Here, transport coefficients have been used to obtain the electronic figure of merit ZT(T) it can be obtained based on the transport coefficients [$\Upsilon_{qq^{'}}$], as:

$$ZT\left( T \right)=\frac{[\Upsilon_{12}]^{2}}{\Upsilon_{11}\Upsilon_{22}-[\Upsilon_{12}]^{2}}$$

**Thermal conductivity and Seebeck coefficient:**

Figure S2(a) displays the thermal conductivity κ(T) of h-BN structures doped with BC dopants. The κ(T) behavior follows a similar pattern to that shown in Fig. 7. For the n4 doped structure at B_T_=0, κ(T) is zero below 2000 K owing to the wide band gap. When a magnetic field is applied and its strength increased, the zero κ(T) region decreases due to band gap reduction. this change is in agreement with the behavior of $\sigma(T)$. The κ(T) becomes larger at higher field strengths since the decreasing band gap from BT allows more electron excitation into the conduction band. For the n_4_-B_C_ structure, κ(T,B_T_=1.8) exhibits the greatest intensity below 2000 K [Fig. S2(a)] because this structure has the zero band gap which allows more charge carriers to be excited into the conduction band.

Figure S2 (b) shows the behavior of the Seebeck coefficient S(T) as a function of temperature for different magnetic field strengths. The results show that at a constant magnetic field strength, the S(T) increases with temperature, and the rate of increase is larger at higher temperatures, especially for smaller magnetic field strengths. In addition, at a constant temperature, the Seebeck coefficient is found to decrease with a reduction in the band gap, which is in agreement with the results of previous studies [3, 4].

**
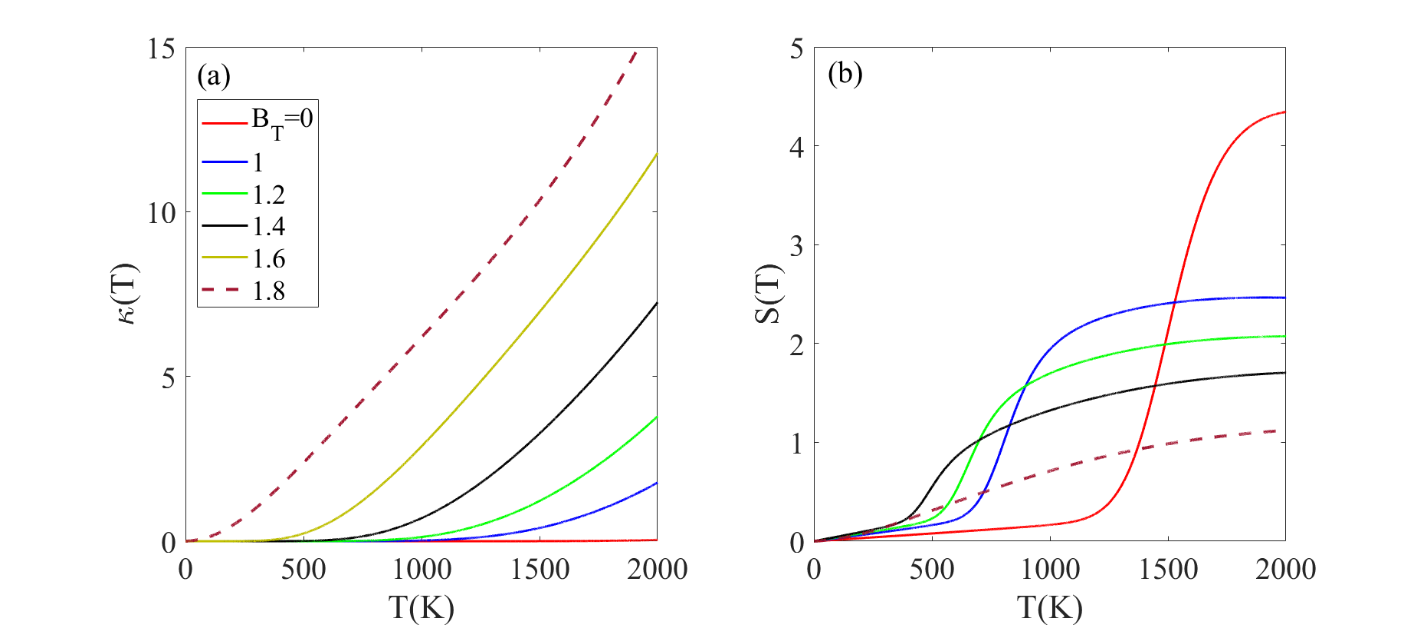
**

**Figure S2**: The temperature dependence of the (a) thermal conductivity and (b) Seebeck coefficient for the n4 doped structure under various magnetic fields. The thermal conductivity increases with increasing magnetic field strengths. At a constant temperature, the Seebeck coefficient decreases as the magnetic field strength is increased.

**Phonon Dispersion:**

Structural optimizations and phonon dispersion calculations were performed using the JDFTx package [5]. The DFT calculations employed a plane-wave basis set with a 35-hartree kinetic energy cutoff. Coulomb truncation was included throughout. During structural optimizations, the atomic positions were relaxed until the force on each atom converged to 0.1 mHa/bohr. To capture the phonon properties, a 3x3x1 supercell was constructed. To prevent interlayer interactions, the sheets were separated by a vacant space of 15 Å. The phonon dispersion calculations demonstrate that hexagonal BN (h-BN) structures with both CB and CN dopant types are dynamically stable, as evidenced by the absence of imaginary phonon frequencies.


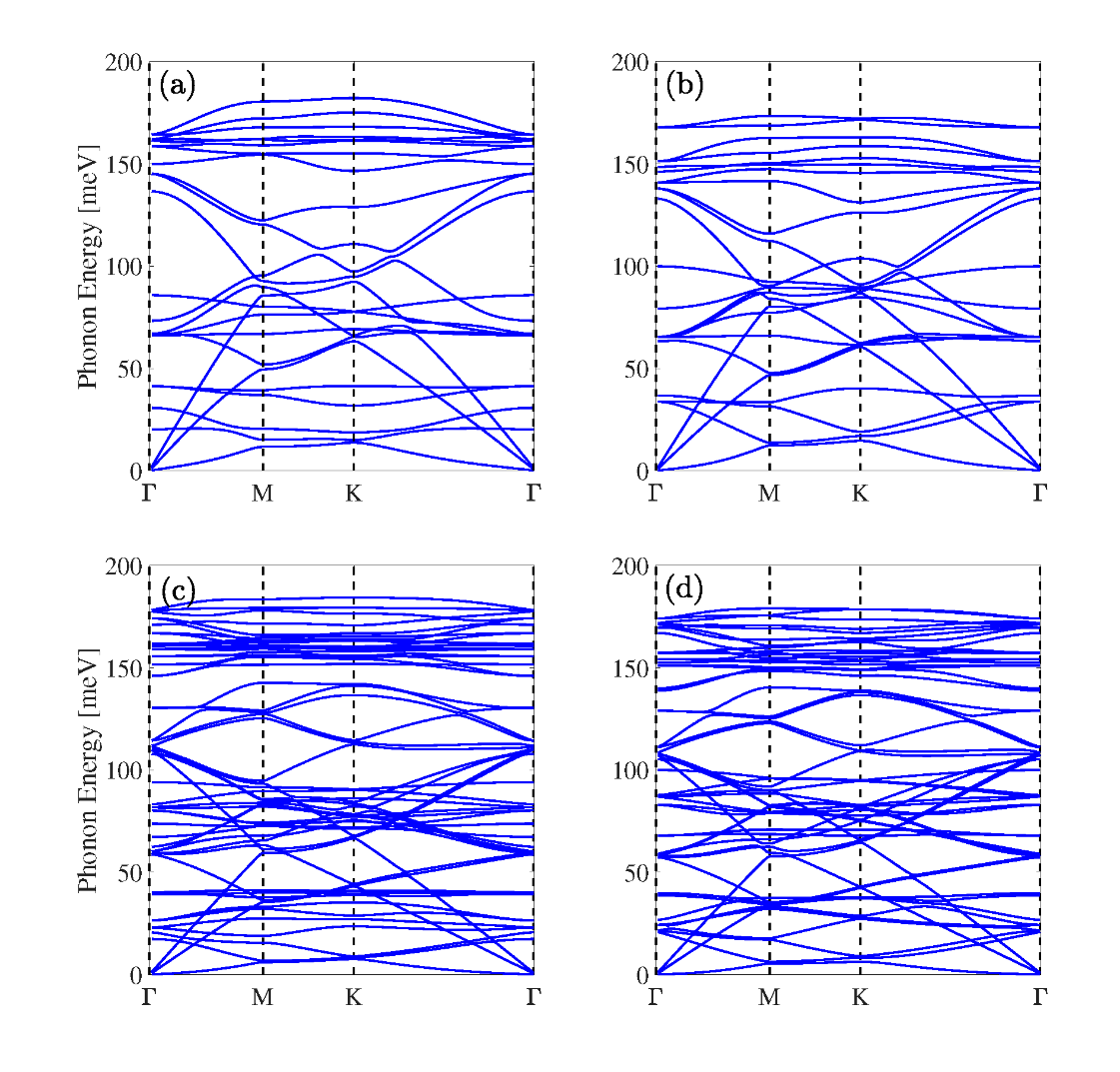


**Figure S3**: The phonon dispersion for n_2_ (a) C_B_ and (b) C_N_ doped structures and for n_3_ (a) C_B_ and (b) C_N_ doped structures, respectively.

[1] J.M. Soler, E. Artacho, J.D. Gale, A. García, J. Junquera, P. Ordejón, D. Sánchez-Portal, The SIESTA method forab initioorder-Nmaterials simulation, Journal of Physics: Condensed Matter, 14 (2002) 2745-2779.

[2] D.M. Ceperley, B.J. Alder, Ground State of the Electron Gas by a Stochastic Method, Physical Review Letters, 45 (1980) 566-569.

[3] A.L. Kutepov, A. Ruth, Electronic structure and thermoelectric properties of CoAsSb with post-DFT approaches, Applied Physics A, 126 (2020) 137.

[4] H.J. Goldsmid, J.W. Sharp, Estimation of the thermal band gap of a semiconductor from seebeck measurements, Journal of Electronic Materials, 28 (1999) 869-872.

[5] S. Grimme, Semiempirical GGA-type density functional constructed with a long-range dispersion correction, Journal of Computational Chemistry, 27 (2006) 1787-1799.
